# Supplementary material for: Predicting visual function by interpreting a neuronal wiring diagram
Source: Nature. 2024 Oct 2;634(8032):113–23. doi: 10.1038/s41586-024-07953-5 (PMC11446822; doi:10.1038/s41586-024-07953-5)
Supplement: Supplementary file 6 — CRF and ERF predictions for individual TmY4 and TmY9 cells. Analogous to Supplementary Data 3, but for TmY target types. Shown are the top four monosynaptic pathways, the strongest pathway passing through each of the top ten intermediary types (ranking from Extended Data Fig. 7), and the trisynaptic pathway Tm1–TmY–Dm3–TmY (see the section entitled Prediction of spatial normalization). [file 41586_2024_7953_MOESM6_ESM.zip › DataS4/TmY9q/720575940628226308.pdf]

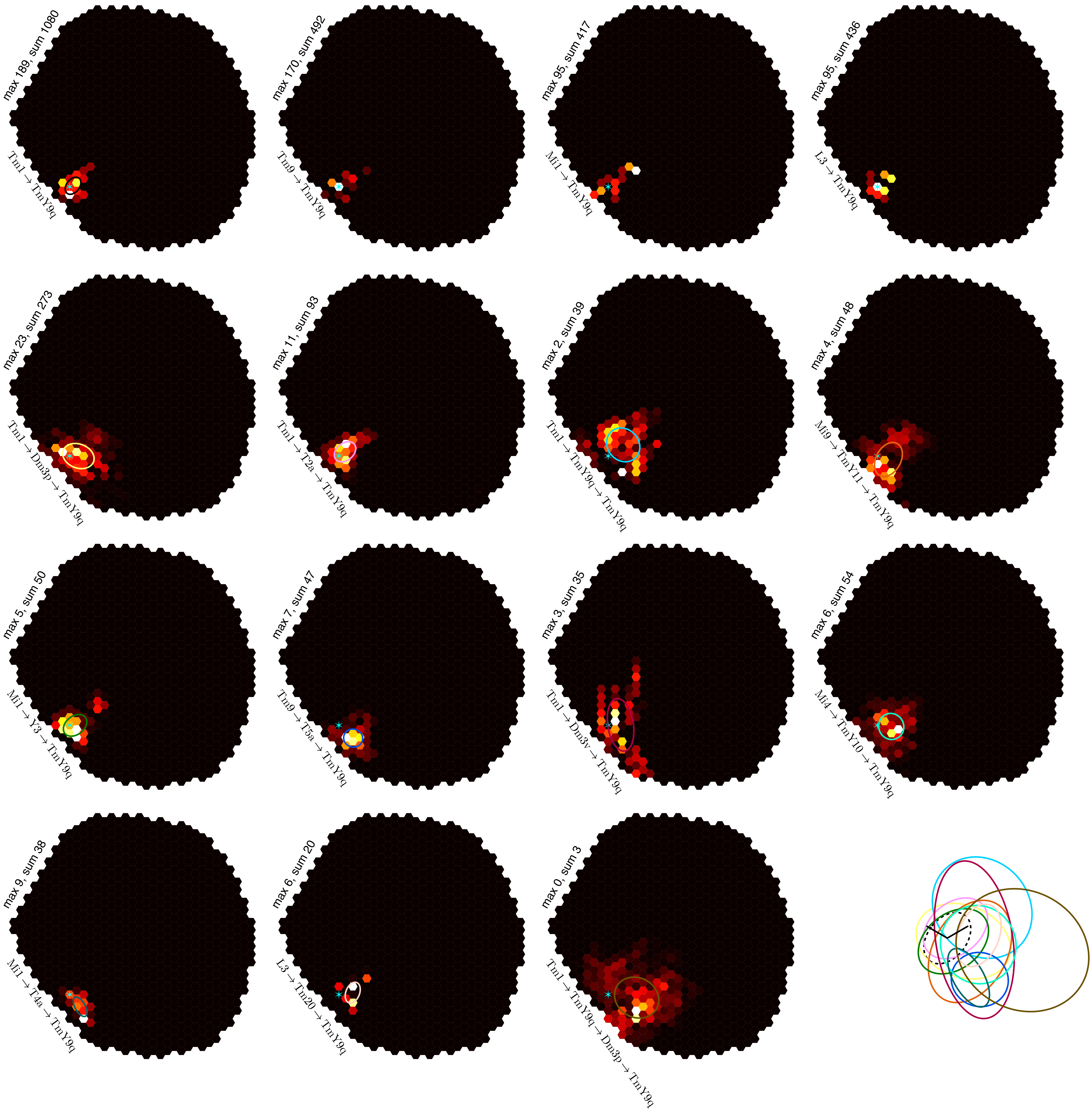
max 189, sum 1080
Tm1 → TmY9q
max 170, sum 492
Tm9 → TmY9q
max 95, sum 417
Mi1 → TmY9q
max 95, sum 436
L3 → TmY9q
max 23, sum 273
Tm1 → Dm3p → TmY9q
max 11, sum 93
Tm1 → T2a → TmY9q
max 2, sum 39
Tm1 → TmY9q → TmY9q
max 4, sum 48
Mi9 → TmY11 → TmY9q
max 5, sum 50
Mi1 → Y3 → TmY9q
max 7, sum 47
Tm9 → T5a → TmY9q
max 3, sum 35
Tm1 → Dm3v → TmY9q
max 6, sum 54
Mi4 → TmY10 → TmY9q
max 9, sum 38
Mi1 → T4a → TmY9q
max 6, sum 20
L3 → Tm20 → TmY9q
max 0, sum 3
Tm1 → TmY9q → Dm3p → TmY9q
